# Association between physical activity and risk of prevalent psoriasis: A MOOSE-compliant meta-analysis

**DOI:** 10.1097/MD.0000000000011394

**Published:** 2018-07-06

**Authors:** Qi Zheng, Xiao Ying Sun, Xiao Miao, Rong Xu, Tian Ma, Ya Nan Zhang, Hong Jin Li, Bin Li, Xin Li

**Affiliations:** a Department of Dermatology, Yueyang Hospital of Integrated Traditional Chinese and Western Medicine, Shanghai University of Traditional Chinese Medicine; b Institute of Dermatology, Shanghai Academy of Traditional Chinese Medicine, Shanghai, China.

**Keywords:** exercise, physical activity, psoriasis, quality of life

## Abstract

Psoriasis is a common chronic relapsing immune-mediated inflammatory disease, whose prevalence has increased in recent years. Some physicians believe that physical activity is associated with numerous health-related benefits in adults with dermatoses. While numerous studies have suggested an association between psoriasis and physical activity, others have yielded contradictory results. The aim of our study was to evaluate the association between the level of physical activity and prevalence of psoriasis.

A comprehensive search of the literature was performed from January 1970 to February 2017 using EMBASE, MEDLINE, and the Cochrane Central Register of Controlled Trials electronic databases. Studies published in English were reviewed to identify the contribution of intensity of physical activity on the prevalence of psoriasis.

The search strategy yielded 1100 relevant studies, among which 13 observational studies were included. We found that patients with psoriasis exercise significantly less vigorously than controls (relative risk [RR]: 0.76; 95% confidence interval [CI]: 0.67–0.85; *P* < .00001). Predominantly, these patients exercised at moderate intensity (RR: 0.40; 95% CI: 0.18–0.90; *P* = .03). Some patients had lesser degree of movement, and some exercised strenuously. There were no significant differences observed in the intensity of exercise performed by controls (RR: 0.90; 95% CI: 0.46–1.77; *P* = .76). The 3 studies found the frequency of regular exercise differed significantly between patients with psoriasis and controls (RR: 0.88; 95% CI: 0.82–0.95; *P* = .0007).

Different severities of psoriasis have different influences on patients’ physical activity levels. Patients with a higher proportion of psoriatic lesions and self-awareness were associated with lower-intensity exercises. Our meta-analysis highlights the fact that intense physical activity may lower the prevalence of psoriasis.

## 1 Introduction

Psoriasis is a common chronic immune-mediated inflammatory disease that affects 2% to 4% of the population in western countries.^[1]^ Psoriasis adversely affects patients’ quality of life, and can also result in a significant financial burden for those affected.^[2]^ The underlying pathogenesis of psoriasis is yet to be completely elucidated. Therefore, conducting appropriate studies and the development of appropriate clinical therapies are key target areas for dermatologists. Recent studies have already shown that moderate-to-severe psoriasis is associated with an increased risk of comorbidities including obesity,^[3,4]^ cardiovascular diseases,^[5]^ type 2 diabetes,^[6]^ hypertension,^[7]^ myocardial infarction,^[8]^ cancer,^[9]^ osteoporosis,^[10,11]^ avascular necrosis,^[12]^ metabolic syndrome,^[13]^ chronic obstructive pulmonary disease (COPD),^[14]^ hyperuricemia,^[15]^ obstructive sleep apnea,^[16]^ and lipid abnormalities.^[17]^

Modern medicine believes that exercising is advantageous to the body. Indeed, long-term moderate exercise can help people feel young and energized. It is commonly known that physical activity is associated with preventive as well as therapeutic health-related benefits for a range of chronic diseases in both patients and the general population. Research has shown that exercise leads to a range of benefits for patients in relation to obesity,^[18]^ cardiovascular disease, musculoskeletal health,^[19,20]^ type 2 diabetes, 20 inflammatory biomarkers,^[21,22]^ cancer,^[23]^ wound healing,^[24]^ and emotional state.^[25]^ Several studies have investigated the relationship between physical activity and psoriasis, and some of these studies have found contradictory data.^[26–30]^ Some scholars believe that physical activity is likely to aggravate the severity of psoriasis, while others believe that psoriasis is likely to improve following a course of voluntary exercise. In our clinic, a large number of patients with psoriatic skin symptoms and mental health problems (anxiety, tension, low self-esteem, isolation, and depression) showed improvements in their condition following varying degrees of physical activity.^[31,32]^ In the present study, we performed a meta-analysis of studies published over the last 47 years that have investigated the correlation between the risk of psoriasis and intensity of physical activity. The purpose of this study was to elucidate the potential underlying association between the intensity of physical activity and the risk of prevalent psoriasis.

## 2 Materials and methods

### 2.1 Data sources and searches

To investigate the relationship between physical activity and psoriasis, 3 reviewers (QZ, XS, and XM) systematically searched EMBASE, MEDLINE, and the Cochrane Central Register of Controlled Trials (CENTRAL) electronic databases for relevant publications using the following keywords: (psoriasis OR physical activity), (psoriasis OR exercise), (psoriasis OR movement), and (psoriasis OR sports). Our search included publications written in English and dated from January 1970 to February 2017.

### 2.2 Study selection

**Figure 1:**
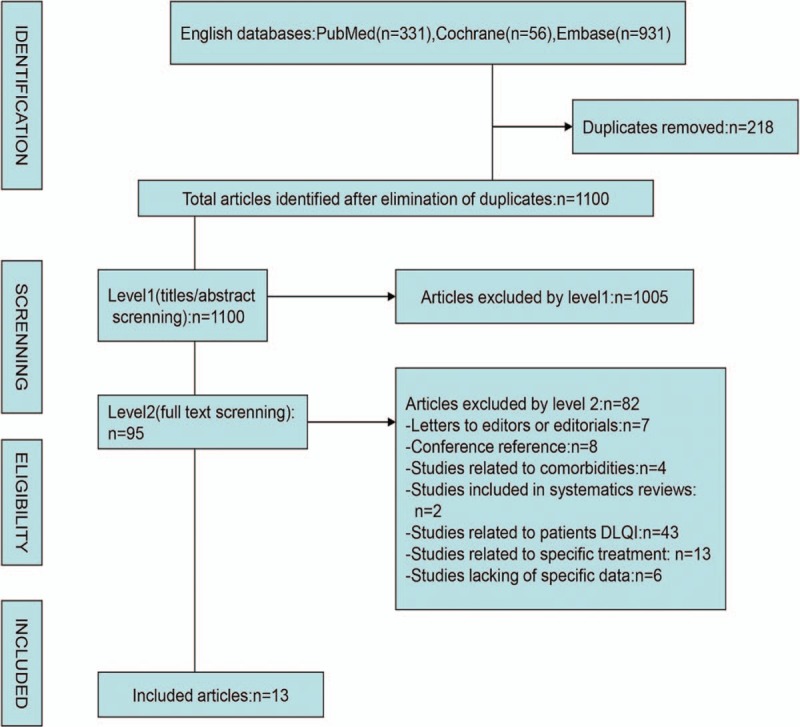
Flowchart depicting the study selection.

In order to identify relevant articles, we screened abstracts for the following criteria: randomized controlled trials and observational studies; studies dedicated to the relationship between psoriasis and physical activity; and studies assessing the impact of physical activity upon quality of life. If an article satisfied these criteria, regardless of the participants’ age, gender, or nationality, we then applied a second phase (Level 2) filter, which purposefully selected articles that were specifically related to humans, and those that featured a significant amount of original data (thus allowing us to extract data for calculation). We deleted 218 duplicated publications, leaving 95 articles that met the selection criteria required by our Level 1 filter. Subsequently, we selected 13 articles that met all the inclusion criteria for the final meta-analysis,^[9,17,33–43]^ as shown in Figure 1 (Table 1).

**Table 1:**
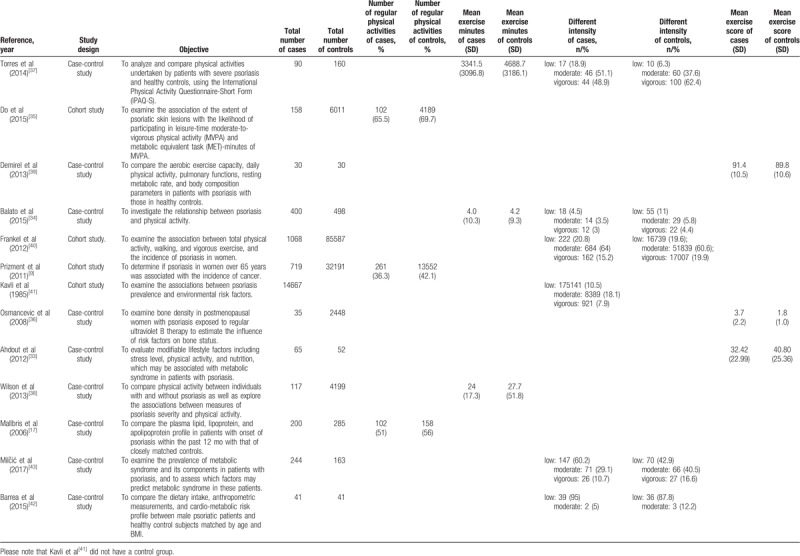
Observational studies included in the meta-analysis.

### 2.3 Data extraction and quality assessment

Descriptive data from each selected study were independently collated by 3 reviewers (QZ, XS, and XM), including the first author; study characteristics (ie, year, objective, and design); participant characteristics (ie, the numbers of patients and controls, and the number of males); and outcome characteristics (ie, exercise minutes and exercise scores for cases and controls, and the frequency of subjects undertaking different intensities of physical activity in the case and control groups). Scores on the Newcastle–Ottawa Scale^[44]^ ranged from 7 to 9, as shown in Table 2. Specifically, 9 studies were regarded as high-quality (8–9 stars) and 4 as medium-quality studies (>6 stars).

**Table 2:**
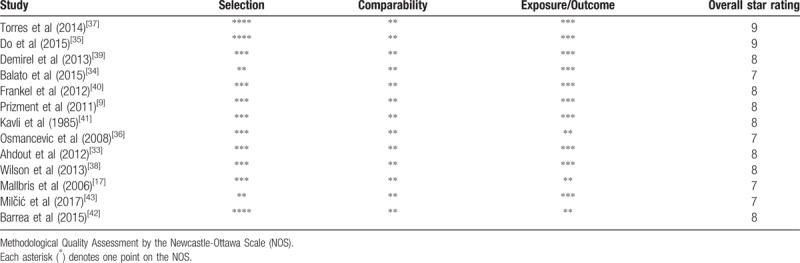
Newcastle–Ottawa Scale (NOS) Quality Assessment Table.

### 2.4 Data synthesis and analysis

The primary outcomes of our analysis of each study were as follows: the different levels of physical activity between healthy controls and patients with psoriasis; and the association between level of physical activity and the risk of psoriasis. Furthermore, we used 2 parameters (exercise minutes and exercise score) as measurable indicators of physical activity. The degree of heterogeneity between the studies was assessed using *I*^2^ tests. An *I*^2^ value >50% was considered to indicate abundant heterogeneity. Randomized-effect models were used to compute standardized mean difference (SMD) and risk ratio (RR) values. Otherwise, when *I*^2^ was <50%, we considered that the study heterogeneity was not substantial and a fixed-effect model was suitable. To investigate the possible explanations for heterogeneity, we performed subgroup analysis using prespecified variables and randomized-effects meta-analysis. Review Manager 5.2 software was used for all meta-analysis (http://ims.cochrane.org/revman).

### 2.5 Ethics and disseminations

Ethical approval is not required in this study because the data used include peer-reviewed publications, which do not comprise any information that could identify subjects.

## 3 Results

This meta-analysis combined data of 13 studies including a total of 149,499 participants. A total of 17,834 patients served as the experimental group, and 131,665 normal people comprised the control group. After merging the relevant data, we divided the intensity exercise into 3 categories: low, moderate, and vigorous.

**Figure 2:**
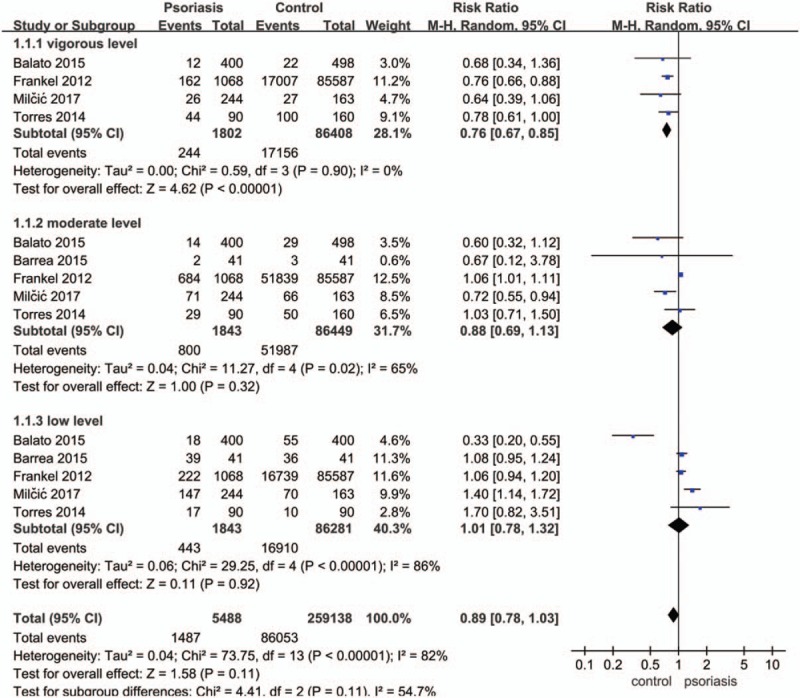
Meta-analysis of the impact on exercise levels of patients with psoriasis and healthy controls. The RR in different exercise levels of psoriatic patients and healthy controls. The point estimate (center of each blue square) and statistical size (proportional area of the square) are represented. Horizontal lines indicate 95% confidence intervals. The subtotal and total pooled RR (diamond) was calculated using a random-effects model. RR = risk ratio.

**Figure 3:**
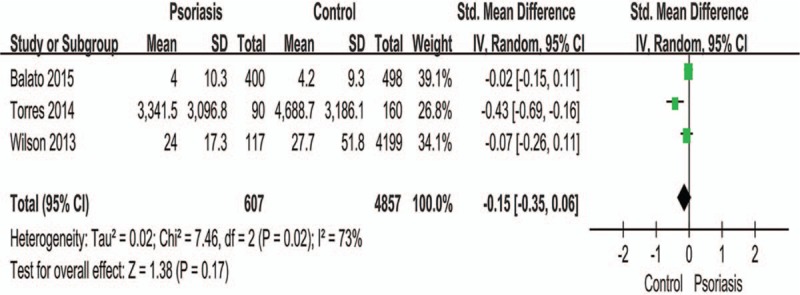
Meta-analysis of the impact on exercise minutes of patients with psoriasis and healthy controls. The SMD in different exercise levels of psoriatic patients and healthy controls. The point estimate (center of each blue square) and statistical size (proportional area of the square) are represented. Horizontal lines indicate 95% confidence intervals. The subtotal and total SMD (diamond) were calculated using a random-effects model. SMD = standard mean difference.

**Figure 4:**
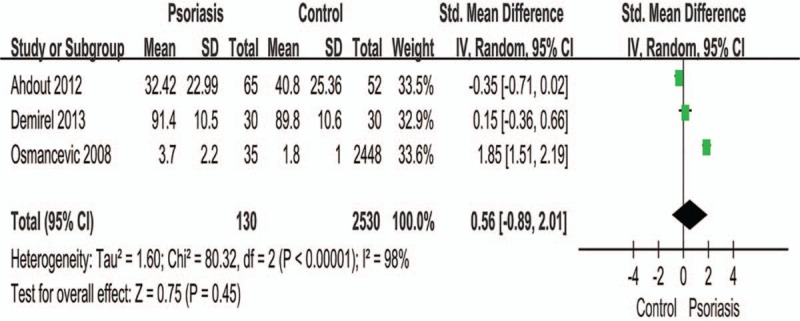
Meta-analysis of the impact on exercise scores of patients with psoriasis and healthy controls. The SMD in different exercise scores of psoriatic patients and healthy controls. The point estimate (center of each blue square) and statistical size (proportional area of the square) are represented. Horizontal lines indicate 95% confidence intervals. The subtotal and total SMD (diamond) were calculated using a random-effects model. SMD = standard mean difference.

There was no significant difference in the level of exercise between people without and with psoriasis when analyzed for the overall effect (Fig. 2) (RR: 0.89; 95% CI: 0.78–1.03; *P* = .11). However, subgroup analysis showed that patients with psoriasis performed vigorous exercises significantly less than controls (RR: 0.76; 95% CI: 0.67–0.85; *P* < .00001). Figures 3 and 4 show that there was no significant difference in terms of exercise duration (SMD: −0.15; 95% CI: 0.36–0.06; *P* = .17) or exercise scores (SMD: 0.56; 95% CI: −0.89 to 2.01; *P* = .44) between patients with psoriasis and controls.

**Figure 5:**
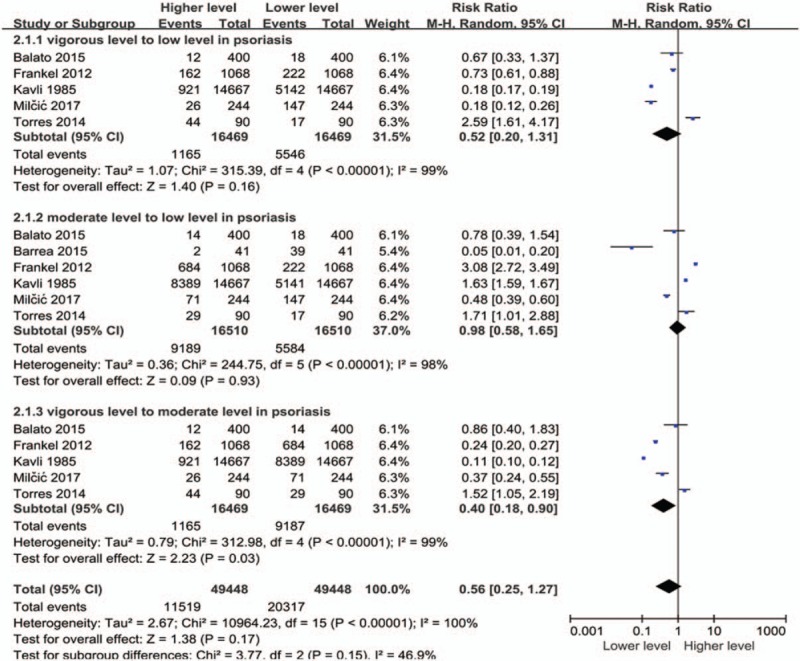
Meta-analysis of patients with psoriasis with different exercise levels. The point estimate (center of each blue square) and statistical size (proportional area of the square) are represented. Horizontal lines indicate 95% confidence intervals. The subtotal and total pooled RR (diamond) was calculated using a random-effects model. RR = risk ratio.

**Figure 6:**
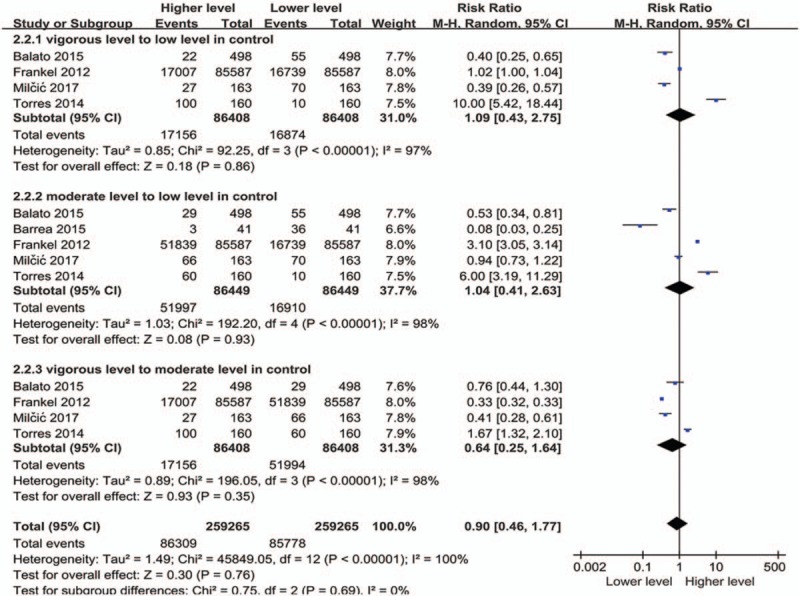
Meta-analysis of healthy controls with different exercise levels. The point estimate (center of each blue square) and statistical size (proportional area of the square) are represented. Horizontal lines indicate 95% confidence intervals. The subtotal and total pooled RR (diamond) was calculated using a random-effects model. RR = risk ratio.

When comparing different intensities of exercise (Fig. 5), it is evident that moderate exercise was the most predominant intensity of exercise in patients with psoriasis (RR: 0.40; 95% CI: 0.18–0.90; *P* = .03). A total of 5584 patients showed a lower degree of movement compared to 1165 patients who carried out strenuous exercises. However, when we compared different intensities of physical activity in people without psoriasis (Fig. 6), we failed to observe any significant relationship; neither in terms of the overall effect (RR: 0.90; 95% CI: 0.46–1.77; *P* = .76) nor in the following subgroup analysis, including a comparison between low, moderate, and vigorous intensities (vigorous vs low, RR: 1.09; 95% CI: 0.43–2.75; *P* = .86; moderate vs low, RR: 1.04: 95% CI: 0.41–2.63; *P* = .93; vigorous vs moderate, RR: 0.64; 95% CI: 0.25–1.64; *P* = .35).

**Figure 7:**
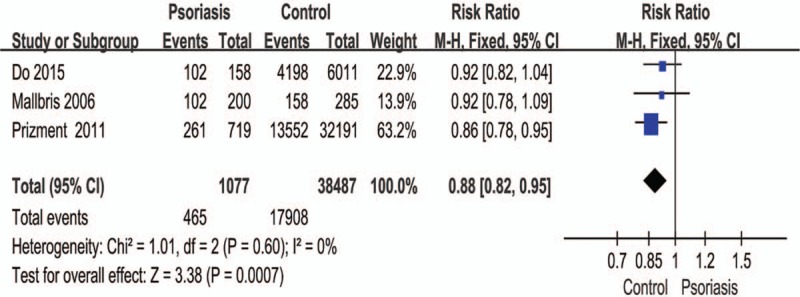
Meta-analysis of the frequency of regular exercise in patients with psoriasis and healthy controls. The point estimate (center of each blue square) and statistical size (proportional area of the square) are represented. Horizontal lines indicate 95% confidence intervals. The subtotal and total pooled RR (diamond) was calculated using a fixed-effects model. RR = risk ratio.

Figure 7 shows that the frequency of regular exercise differed significantly between patients with psoriasis and controls (RR: 0.88; 95% CI: 0.82–0.95; *P* = .0007). By comparing the daily amount of regular exercising performed before suffering from psoriasis, we found that the number of patients was lower than the controls.

## 4 Discussion and conclusions

Psoriasis is a recurrent incurable inflammatory disease with complex etiology. Prospective studies^[3]^ have demonstrated that infections, immune dysfunctions, metabolic disorders, and emotional fluctuations are pathogenic factors in psoriasis. In additional, alcohol intake,^[45]^ smoking,^[46]^ obesity,^[47]^ and fatigue^[48]^ are risk factors of psoriasis. However, relatively fewer studies have focused on identifying protective factors for psoriasis. Exercise is one such factor, but the relationship between exercise and psoriasis remains to be further explored. Numerous studies have proven that psoriasis is closely associated with physical activity. The theory that only vigorous physical activity can decrease the risk of incident psoriasis was introduced by Frankel.^[40]^ Other studies have shown that the level of physical activity could influence the prevalence of psoriasis. Lewis-Beck et al^[30]^ conducted a questionnaire survey among 199 patients with moderate-to-severe psoriasis and showed that 72.36% of patients reported impaired levels of activity because of psoriasis. Another study^[26]^ reported that sporting activities were significantly (*P* < .001) affected among patients with mild, moderate, and severe psoriasis. Additionally, in a study by Leino et al,^[28]^ 23.7% of patients had reduced their sporting activities while 30.2% had stopped completely. In accordance with these results, our meta-analysis exposes the relationship between the degree of exercise and prevalence of psoriasis.

As shown by the large number of articles published in recent years, the negative effects of moderate-to-severe psoriasis on both work and physical activity are becoming increasingly credible. Our current meta-analysis extended the data of the earlier studies by investigating the correlation between psoriasis and physical activity. The main contents of the included studies were about the prevalence of psoriasis except for that of Frankel,^[40]^ which aimed to interpret the association of physical activity and risk of incident psoriasis. Although prevalence rate and incidence rate are different, prevalence rate is dependent on the incidence rate and duration. As the incidence rate increases, the rate of prevalence increases correspondingly. Therefore, we included this study into our meta-analysis. We recruited a group of patients with psoriasis (n = 17834), and another group of healthy subjects without psoriasis (n = 131665). Then, we compared the two groups directly and analyzed subgroups by using a range of data extracted from the previous publications identified, including exercise duration, exercise score (the Godin Leisure-Time Exercise Questionnaire and Quality of life questionnaire: SF-36) and the frequency of regular exercising in the past. We classified the intensity of exercise into low, moderate, and vigorous. Our results showed a causal link between physical activity and prevalence of psoriasis. Psoriasis will reduce vigorous exercising and only vigorous physical activity will decrease the prevalence of psoriasis. Furthermore, we believe that strenuous physical activity is related to a reduction in the prevalence of psoriasis. Otherwise, regular bouts of physical activity can improve psoriatic skin lesions due to the benefits of sunlight during outdoor exercises.^[49]^

It is difficult to precisely interpret the data shown in Figure 4, which illustrates the differences in exercise duration between patients with psoriasis and normal controls (RR: 0.56; 95% CI: −0.89 to 2.01; *P* = .45). This is because exercise time cannot be directly translated into the intensity of the physical activity, for example, longer exercise duration does not necessarily indicate a higher severity of exercise. Exercise intensity refers to the degree of physical stimulation of the body. A previous study^[39]^ used a questionnaire or an analogue scale, which uses a combination of physical and mental health scores, with higher scores indicating better function. The authors of that study believed that patients with mild-to-moderate psoriasis had a lifestyle that involved physical activity. We understand that patients with psoriasis tend to exercise more than the controls, and this may be attributed to patients receiving advice from physicians regarding moderate exercise and therefore have increased awareness of the significance of exercise.

Finally, it is important to note that traditional meta-analyses are often limited by confounding factors, such as heterogeneity and publication bias. The diversity of designs and the inherent bias of observational studies make it challenging to examine the methodological quality. Because we analyzed a relatively small number of publications in our meta-analysis, there is a high risk of statistical heterogeneity (*I*^2^ > 50%). In addition, factors such as obesity, age, gender, and economic conditions will affect patients’ ability to exercise and therefore these should be considered as potential bias in this paper. In order to reduce this risk, we performed subgroup analyses. Regardless of potential publication bias, we applied strict inclusion and exclusion criteria, which should reduce the impact of such bias. Therefore, the aforementioned potential limitations must be considered when interpreting the study conclusions.

## Author contributions

**Conceptualization:** Rong Xu, Xin Li.

**Data curation:** Qi Zheng, Ya Nan Zhang.

**Formal analysis:** Xiao Ying Sun, Xiao Miao, Tian Ma.

**Investigation:** Xin Li.

**Methodology:** Rong Xu.

**Project administration:** Bin Li.

**Software:** Tian Ma, Ya Nan Zhang.

**Supervision:** Xiao Miao.

**Validation:** Qi Zheng.

**Writing – original draft:** Qi Zheng, Xiao Ying Sun.

**Writing – review & editing:** Hong Jin Li, Bin Li, Xin Li.
